# 

**Published:** 1903-06-27

**Authors:** 


### NOTICE TO CORRESPONDENTS.

All MSS., Letters, Books for Review, and other matters intended for the Editor should be addressed The Editor, "The Hospital," The Hospital Buildings, 28 & 29 Southampton Street, Strand, W.C.

The Editor cannot undertake to return rejected MS., even when accompanied by stamped directed envelope.

**Notice to Advertisers and Subscribers.**

All Advertisements, Orders for Copies of The Hospital, and Business Communications should be addressed to **THE MANAGER, (Not to the Editor)**, The Hospital, 28 & 29 Southampton Street, Strand, London, W.C.

The Cost of Subscription, post free, is as follows.—Per week, 3½d.; per quarter, 3s. 9d.; per half-year, 7s. 6d.; per annum, 15s. Foreign and Colonial:—Per annum, 19s. 6d., payable, in all cases, in advance.

**NOTICE.—Vol. XXXIII. of "The Hospital" (October 1902 to March 1903) in handsome cloth binding, will shortly be on sale at the office of this Journal, price 7s. 6d. Cases for binding the Numbers contained in this Volume 1s. 6d. Index to Vol. XXXIII., 2½d., post free.**

**The monthly part for July will be ready on July 1. Price 1s., by post 1s. 3d.**

### The Student of Medicine.

### Appointments.

**W. Ball**, L.S.A., appointed Second Assistant Medical Officer of the St. George's Union Infirmary. **W. N. Barron**, M.R.C.S., L.R.C.P.Lond., appointed District Medical Officer of the East-hampstead Union. **J. Lindley Carstairs**, M.A., M.B., C.M.Glasg., appointed Outdoor Physician for North-Western District, Glasgow Maternity Hospital. **J. B. Cook**, M.B., Ch.B.Vict., M.R.C.S., L.R.C.P., appointed House Surgeon to the Royal Alexandra Hospital for Sick Children, Brighton. **Myer Coplans**, M.B.Lond., appointed Resident Medical Officer to the North-West London Hospital. **Henry Davis**, M.R.C.S., appointed Anæsthetist to the French Hospital. **C. C. J. Erhardt**, M.R.C.S., L.R.C.P.Lond., appointed District Medical Officer of the Skipton Union. **Leonard M. Fleetwood**, L.D.S.R.C.S.Eng., appointed Honorary Surgeon-Dentist to the Chichester Infirmary. **R. Brennand Fletcher**, M.B., Ch.B.Vict., appointed Resident Medical Officer to St. Mary's Hospital, Manchester. **G. O. Gauld**, M.B., B.S.Aberd., appointed District Medical Officer of the Pocklington Union. **Harry Harlock**, L.R.C.P.Lond., M.R.C.S.Eng., appointed Physician to the Chichester Infirmary. **H. Willoughby Lyle**, M.D., B.S.Lond., F.R.C.S.Eng., appointed Honorary Assistant Surgeon to the Royal Eye Hospital, Southwark. **Professor Antony Roch**, M.R.C.P., L.R.C.S.Irel., appointed Lecturer on Hygiene to the Department of Technical Education and Agriculture for Ireland. **J. W. Stephens**, M.R.C.S., L.R.C.P.Lond., appointed District and Workhouse Medical Officer of the Cardigan Union. **R. R. Sutter**, M.D., M.S.Aberd., appointed District Medical Officer of the St. Ives Union. **A. F. Thompson**, M.B., Ch.B.Vict., appointed House Surgeon to the Manchester Royal Infirmary. **Winifred Thorp**, M.B., B.S.Lond., appointed House Surgeon to the Kettering General Hospital. **G. R. Young**, M.D., M.Ch.R.U.I., appointed Medical Officer of the Langley House Receiving Home of the Poplar Union.

### Books for Private Nurses.

### ART OF MASSAGE.

By Mrs. CREIGHTON HALE.

Price **6/-** post free.

Every Nurse should master the "Art of Massage." She will find it useful and remunerative. Mrs. Creighton Hale who has had perhaps more experience as a teacher than any other Masseuse has been able in her book to explain with great clearness all the difficulties the pupil meets with.

### OPHTHALMIC NURSING.

By SYDNEY STEPHENSON.

Price **3/6** net, **3/10** post free.

This is a complete and profusely-illustrated work, which should be in the hands of all Nurses in charge of Eye cases.

### THE CARE OF CONSUMPTIVES.

By W. H. DAW, M.R.C.S.

Price **1/-** post free.

The hygienic treatment of consumptives is now regarded as so important that the trained nurse plays a very necessary part in the care of these invalids. The subject cannot be fully studied in the general hospital, therefore every nurse should add to her knowledge by the use of this admirable little text-book.

### FEVERS AND INFECTIOUS DISEASES.

By Dr. WILLIAM HARDING.

Price **1/-** post free.

This is a valuable introduction to the nursing and practical management of these cases.

### THE POCKET CASE-BOOK.

Price **1/-** post free.

Nurses will find this a concise and convenient little case-book.

### ON PREPARATION FOR OPERATION IN PRIVATE HOUSES.

By STANMORE BISHOP, F.R.C.S.

Price **6d.** post free.

A useful little help and guide to the nurse engaged for an operation case in a private house.

*These books are obtainable of all Booksellers, and are published by*
THE SCIENTIFIC PRESS, LIMITED, 28 & 29 Southampton Street, Strand, London,
*who will send their latest Catalogue of Nursing Text-books post free to any Nurse on application.*

